# SAkuraBONSAI: Protocol design of a novel, prospective study to explore clinical, imaging, and biomarker outcomes in patients with AQP4-IgG-seropositive neuromyelitis optica spectrum disorder receiving open-label satralizumab

**DOI:** 10.3389/fneur.2023.1114667

**Published:** 2023-02-17

**Authors:** Jeffrey L. Bennett, Kazuo Fujihara, Ho Jin Kim, Romain Marignier, Kevin C. O'Connor, Robert C. Sergott, Anthony Traboulsee, Heinz Wiendl, Jens Wuerfel, Scott S. Zamvil, Veronica G. Anania, Regine Buffels, Thomas Künzel, Annemarie N. Lekkerkerker, Siân Lennon-Chrimes, Sean J. Pittock

**Affiliations:** ^1^Programs in Neuroscience and Immunology, Departments of Neurology and Ophthalmology, University of Colorado Anschutz Medical Campus, Aurora, CO, United States; ^2^Department of Multiple Sclerosis Therapeutics, Fukushima Medical University School of Medicine, Multiple Sclerosis and Neuromyelitis Optica Center, Southern TOHOKU Research Institute for Neuroscience, Koriyama, Japan; ^3^Department of Neurology, National Cancer Center, Goyang, Republic of Korea; ^4^Service de Neurologie, Sclérose en Plaques, Pathologies de la Myéline et Neuroinflammation, Centre de Référence des Maladies Inflammatoires Rares du Cerveau et de la Moelle, Hôpital Neurologique Pierre Wertheimer, Hospices Civils de Lyon, Lyon, France; ^5^Departments of Neurology and Immunobiology, Yale University School of Medicine, New Haven, CT, United States; ^6^Annesley EyeBrain Center, Wills Eye Hospital, Thomas Jefferson University, Philadelphia, PA, United States; ^7^Department of Medicine (Neurology), University of British Columbia, Vancouver, BC, Canada; ^8^Department of Neurology, University of Muenster, Münster, Germany; ^9^Medical Image Analysis Centre (MIAC AG) and University of Basel, Basel, Switzerland; ^10^F. Hoffmann-La Roche Ltd, Basel, Switzerland; ^11^Department of Neurology and Program in Immunology, University of California, San Francisco, San Francisco, CA, United States; ^12^Genentech, Inc., San Francisco, CA, United States; ^13^Roche Products Ltd, Hertfordshire, United Kingdom; ^14^Department of Neurology, Center for MS and Autoimmune Neurology, Mayo Clinic, Rochester, MN, United States

**Keywords:** biomarkers, magnetic resonance imaging (MRI), neuromyelitis optica spectrum disorder (NMOSD), optical coherence tomography (OCT), satralizumab, relapse

## Abstract

**Background:**

Neuromyelitis optica spectrum disorder (NMOSD) is a rare, autoimmune disease of the central nervous system that produces acute, unpredictable relapses causing cumulative neurological disability. Satralizumab, a humanized, monoclonal recycling antibody that targets the interleukin-6 receptor, reduced NMOSD relapse risk vs. placebo in two Phase 3 trials: SAkuraSky (satralizumab ± immunosuppressive therapy; NCT02028884) and SAkuraStar (satralizumab monotherapy; NCT02073279). Satralizumab is approved to treat aquaporin-4 IgG-seropositive (AQP4-IgG+) NMOSD. SAkuraBONSAI (NCT05269667) will explore fluid and imaging biomarkers to better understand the mechanism of action of satralizumab and the neuronal and immunological changes following treatment in AQP4-IgG+ NMOSD.

**Objectives:**

SAkuraBONSAI will evaluate clinical disease activity measures, patient-reported outcomes (PROs), pharmacokinetics, and safety of satralizumab in AQP4-IgG+ NMOSD. Correlations between imaging markers (magnetic resonance imaging [MRI] and optical coherence tomography [OCT]) and blood and cerebrospinal fluid (CSF) biomarkers will be investigated.

**Study design:**

SAkuraBONSAI is a prospective, open-label, multicenter, international, Phase 4 study that will enroll approximately 100 adults (18–74 years) with AQP4-IgG+ NMOSD. This study includes two patient cohorts: newly diagnosed, treatment-naïve patients (Cohort 1; *n* = 60); and inadequate responders to recent (<6 months) rituximab infusion (Cohort 2; *n* = 40). Satralizumab monotherapy (120 mg) will be administered subcutaneously at Weeks 0, 2, 4, and Q4W thereafter for a total of 92 weeks.

**Endpoints:**

Disease activity related to relapses (proportion relapse-free, annualized relapse rate, time to relapse, and relapse severity), disability progression (Expanded Disability Status Scale), cognition (Symbol Digit Modalities Test), and ophthalmological changes (visual acuity; National Eye Institute Visual Function Questionnaire-25) will all be assessed. Peri-papillary retinal nerve fiber layer and ganglion cell complex thickness will be monitored using advanced OCT (retinal nerve fiber layer and ganglion cell plus inner plexiform layer thickness). Lesion activity and atrophy will be monitored by MRI. Pharmacokinetics, PROs, and blood and CSF mechanistic biomarkers will be assessed regularly. Safety outcomes include the incidence and severity of adverse events.

**Conclusions:**

SAkuraBONSAI will incorporate comprehensive imaging, fluid biomarker, and clinical assessments in patients with AQP4-IgG+ NMOSD. SAkuraBONSAI will provide new insights into the mechanism of action of satralizumab in NMOSD, while offering the opportunity to identify clinically relevant neurological, immunological, and imaging markers.

## 1. Introduction

Neuromyelitis optica spectrum disorder (NMOSD), a rare, autoimmune, neurological disorder, produces inflammatory lesions in the optic nerve, spinal cord, brainstem, and cerebrum (1–3). Serum immunoglobulin G (IgG) autoantibodies that target the water channel aquaporin-4 (AQP4) are a highly specific, pathogenic biomarker of NMOSD (4).

People with NMOSD experience unpredictable, severe attacks of optic neuritis and/or longitudinally extensive transverse myelitis, which can cause potentially severe motor and sensory impairment, bladder dysfunction, vision loss, pain, and other debilitating neurological symptoms (1–3).

Because a single relapse can cause significant, lasting disability, maintenance treatment to reduce the frequency and severity of attacks has become standard practice. Satralizumab is a humanized, IgG2 subclass, monoclonal recycling antibody that targets the soluble and membrane-bound form of the interleukin (IL)-6 receptor (IL-6R) (5–7). In the pivotal Phase 3 SAkuraSky and SAkuraStar studies, satralizumab significantly reduced the risk of NMOSD relapse in AQP4-IgG+ patients by 79 and 74%, respectively, compared with placebo (6, 7). Based on these results, satralizumab is now approved in more than 60 countries for the treatment of AQP4-IgG+ NMOSD, including Canada, Japan, the United States, and the European Union (8–10).

Anti-CD20 agents such as rituximab (RTX) are among the most commonly used maintenance therapies in NMOSD (11), but some individuals remain inadequately controlled despite treatment (12, 13). Both SAkuraSky and SAkuraStar excluded patients who had been treated with RTX in the prior 6 months, resulting in the enrollment of fewer than 20 patients with historic RTX use. Patients with an inadequate response to RTX warrant further study to examine their disease status and profile at baseline, as some patients with NMOSD who were unresponsive to anti-CD20 treatment have demonstrated clinical benefit when treated with IL-6R antagonist therapy (14). Additional research is also required to improve our understanding of the effects of early intervention with satralizumab in newly diagnosed, treatment-naïve patients.

There are no established prognostic markers for disease activity in NMOSD, making periods of relapse or stability unpredictable. Key questions remain on the optimal use of magnetic resonance imaging (MRI) and optical coherence tomography (OCT) techniques, including the potential for subclinical “silent” disease activity or progression independent of relapse activity. Serum levels of glial acidic protein (GFAP) and neurofilament light chain (NfL), which are intermediate filament protein markers of astrocytes and neurons, respectively, have previously been suggested to be potential biomarkers of disease activity and disability in NMOSD (15–17). Other markers of immune-related activity, such as IL-6, IL-17A, and the terminal complex of the complement cascade, could also provide insight into the pathophysiology and prognosis of NMOSD (18).

Here, we report the study design of SAkuraBONSAI, a prospective study to assess comprehensive longitudinal, clinical, imaging, and blood and cerebrospinal fluid (CSF) mechanistic biomarker assessments in NMOSD. SAkuraBONSAI will advance our understanding of NMOSD disease progression and treatment response in patients who are newly diagnosed and treatment-naïve, and in individuals who have responded inadequately to RTX.

## 2. Methods and analysis

### 2.1. Objectives

The primary objective of this study is to describe the efficacy of satralizumab in patients with AQP4-IgG+ NMOSD who are treatment-naïve or inadequate responders to previous treatment with RTX or its biosimilars. The secondary objective is to describe the evolution of advanced imaging outcomes over time in these patients, including MRI analyses of the entire central nervous system (CNS; brain, optic nerve, and spinal cord), and OCT assessments of changes in the thickness of the retinal nerve fiber layer (RNfL) and the ganglion cell plus inner plexiform layer (GCIPL). Exploratory objectives include the impact of satralizumab on patient-reported outcomes (PROs) and biomarkers relevant to NMOSD pathophysiology (including immune and CNS-related neuronal biomarkers), and baseline and longitudinal correlations between CSF, circulating biomarkers, and clinical and imaging measures. The pharmacokinetics (PK) of satralizumab in CSF and serum, and the immunogenicity of satralizumab, will also be assessed. Safety endpoints will assess the incidence and severity of adverse events (AEs), plus vital signs and clinical laboratory tests.

### 2.2. Study design

**Figure 1:**
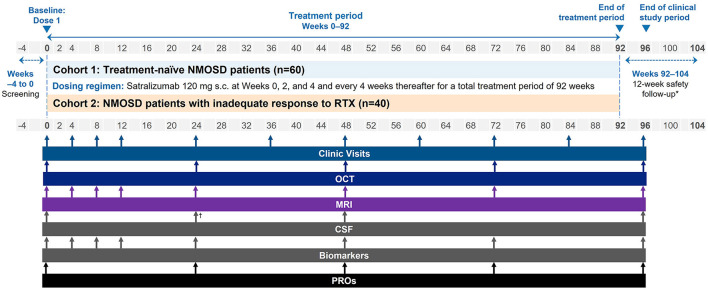
Study schema. *For participants withdrawing from satralizumab treatment, i.e. those discontinuing early or not continuing with treatment outside of the study. ^†^Optional CSF at Week 24. CSF, cerebrospinal fluid; MRI, magnetic resonance imaging; NMOSD, neuromyelitis optica spectrum disorder; OCT, optical coherence tomography; PRO, patient-reported outcome; RTX, rituximab; s.c., subcutaneous.

SAkuraBONSAI is a Phase 4, prospective, multicenter, open-label, efficacy and safety study of satralizumab in patients with AQP4-IgG+ NMOSD (Figure 1). The study will enroll 100 participants across two cohorts. Cohort 1 will consist of patients who are treatment-naïve with no history of disease-modifying or immunosuppressive therapy (*n* = 60). Cohort 2 will comprise patients who have been treated with RTX and responded inadequately (*n* = 40).

Clinical visits and imaging and fluid biomarker assessments will be performed regularly according to the schedule in Figure 1. At Week 0, baseline clinical, laboratory, and imaging assessments will be performed at the study site. Frequent (approximately every 4 weeks) clinical, MRI, and other biomarker assessments will take place in the first 12 weeks of the trial to follow in detail the onset of the satralizumab treatment effect. A clinical evaluation will then take place at Week 96, followed by a 12-week safety follow-up.

### 2.3. Study treatments and at-home dosing

Participants will be treated with satralizumab 120 mg as monotherapy via subcutaneous injection using a pre-filled syringe at Weeks 0, 2, 4, and then every 4 weeks until the last administration at Week 92. This is consistent with the licensed dosing regimen of satralizumab in AQP4-IgG+ NMOSD (9, 10). The first dose of satralizumab will be administered by the site staff at Week 0. The second dose will be self-administered by participants (or their caregivers) under the supervision of a designated study staff member at the study site at Week 2. If the treating physician is satisfied that the participant (or their caregiver) can perform the injection, all subsequent doses may be administered at home. A telephone interview the working day after each satralizumab dose will confirm compliance, and evaluate any changes in health status (e.g., new or worsening neurological symptoms, or any possible AEs).

Rescue therapy for clinical relapses and pain medications are both permitted during the study. Rescue therapies include pulse intravenous corticosteroids, oral corticosteroids for tapering, intravenous immunoglobulin, and/or apheresis (including plasma exchange [PLEX] and plasmapheresis). Pain medications include, but are not limited to, pregabalin, gabapentin, carbamazepine, clonazepam, duloxetine, and tramadol/acetaminophen. Combination treatment with immunosuppressive therapies (e.g., azathioprine, cyclosporine, methotrexate, mycophenolate mofetil, tacrolimus) is not permitted, even if a relapse occurs.

### 2.4. Eligibility criteria

Eligible participants will be 18–74 years old with AQP4-IgG+ NMOSD according to the International Panel for Neuromyelitis Optica Diagnosis criteria (4), confirmed by central AQP4-IgG+ testing. After providing informed consent, participants will enter a screening period of up to 4 weeks. Participants treated with oral corticosteroids must taper to steroid-free at least 1 week before initiating satralizumab; these patients are permitted a prolonged total screening period of up to 5 weeks.

To be included in Cohort 1 (treatment-naïve), patients must have clinical evidence of at least one documented NMOSD attack or relapse (including first attack) within 12 months of screening and be naïve to disease-modifying or immunosuppressive maintenance therapy. Acute relapse rescue therapy (e.g., intravenous steroid pulse, PLEX, or plasmapheresis) is permitted.

Cohort 2 (inadequate RTX responders) will include patients with an NMOSD disease duration ≤5 years who have responded inadequately to RTX or its biosimilar. They must have received ≥2 RTX courses (a course is defined as 2 infusions dosed 14 days apart), with the most recent course within 6 months of screening; have ongoing disease activity (relapse and/or any new inflammatory event, confirmed by MRI or ophthalmological assessment) after the last course; and not have discontinued RTX treatment for any reason other than inadequate response.

Key study-wide exclusion criteria include: being unable to undergo MRI assessments, pregnancy or breastfeeding, evidence of diseases that may preclude participation (e.g., other demyelinating disease, neurologic/ophthalmologic conditions, neoplastic disease, or infection), prior treatment with any investigational agent ≤6 months prior to baseline (or 5 drug elimination half-lives of the investigational agent), leukopenia, neutropenia, lymphocytopenia, thrombocytopenia, or elevated aspartate aminotransferase or alanine aminotransferase levels at baseline.

### 2.5. Medical history and baseline assessments

At baseline, patients will undergo a full physical and neurological examination (including Expanded Disability Status Scale [EDSS] score), MRI scan, and OCT examination. Patient demographics will be gathered, including age, gender, and self-reported race/ethnicity. Individuals' NMOSD disease history will be evaluated, including the date, symptoms, and severity of first symptom, and the number of relapses in the past year (including description of relapse and symptoms). For Cohort 2, treatment history (including start and stop dates and reason for change in treatment) and RTX antibody status and level (to understand the reason for RTX treatment failure) will be gathered.

### 2.6. Relapse assessment

Clinical relapses will be reported and assessed against the criteria for protocol-defined relapse (Table 1). In the event of a suspected relapse, patients are required to visit the study site within 7 days for an EDSS/Functional System Score assessment, performed by an appropriately qualified assessor, plus a complete set of MRI, OCT, and fluid biomarker assessments.

**Table 1:** Protocol-defined relapse criteria.

**The following criteria must be met for a protocol-defined relapse:**
• New or worsening neurological symptoms compared with the most recent scheduled visit, meeting any of the following criteria:
° ≥1.0-point increase in EDSS score (a ≥2.0-point increase is required if the baseline score was zero)
° ≥2.0-point increase in one of the appropriate FSSs
° ≥1.0-point increase on two or more of the appropriate FSSs if the baseline score was ≥1.0
° ≥1.0-point increase in single-eye FSS when the baseline score in that eye was ≥1.0
• Symptoms must persist for >24 h and should be attributable to NMOSD, and not confounding clinical factors (e.g., fever, infection, injury, change in mood, adverse reactions to medications)
• New or worsening neurological symptoms that occur <31 days following the onset of a relapse will be considered as part of the same relapse (i.e., a flare-up)

EDSS, Expanded Disability Status Scale; FSS, Functional System Score; NMOSD, neuromyelitis optica spectrum disorder.

### 2.7. Disability assessments

EDSS will be used as a quantitative measure of disability and for the assessment of relapse severity. Visual acuity will be assessed using high-contrast (100%) and low-contrast (2.5%) visual acuity at distances of both 4 and 1 meters. Best corrected high-contrast visual acuity will be tested with the standard Early Treatment Diabetic Retinopathy Study (ETDRS) charts. Low-contrast visual acuity will be tested with 2.5% Sloan charts. Routine refraction assessments will be conducted at the start of the study. The National Eye Institute Visual Function Questionnaire-25 (NEI VFQ-25) will be used to assess a participant's perception of vision-related functioning and vision-related quality of life.

### 2.8. Imaging assessments

MRI will be used to monitor focal CNS lesions and may be used to assess other pathophysiology, such as diffuse inflammation and neurodegeneration. In this multisite trial with harmonized protocols, MRI acquisition comprises isotropic sagittal 3D naive and contrast enhanced T1w, isotropic sagittal 3D FLAIR, and GRE QSM, as well as axial 2D multiband DTI, contrast-enhanced DSC, and orbital coronal 2D fat-saturated T2w and contrast-enhanced T1w MRI for brain imaging; sagittal 2D T2w and contrast enhanced T1w whole spinal cord MRI as well as axial T2w and contrast enhanced T1w MRI covering spinal cord lesions. MRI output data will be assessed by a centralized reading center for efficacy endpoints. MRI parameters of the brain, optic nerve, and spinal cord are shown in Table 2. OCT assessments will include change in the RNfL thickness and change in the GCIPL thickness. Spectral domain OCT will be performed using the Heidelberg SPECTRALIS^®^ imaging platform (with or without Glaucoma Module Premium Edition [GMPE]/Anatomic Positioning System [APS]) or Zeiss CIRRUS^®^ OCT based on site machine availability. Multicolor OCT will be performed at selected sites. OCT measurements will be compared based on the device and software application used. Advised Protocol for OCT Study Terminology and Elements (APOSTEL) 2.0 recommendations and OSCAR-IB criteria will be used for reporting and quality control (19, 20).

**Table 2:** MRI assessments in SAkuraBONSAI.

**MRI assessments of the brain and optic nerves will include (but are not limited to):**
• Count, volume, and regional distribution of T2-weighted fluid-attenuated inversion recovery (FLAIR) hyperintense lesions, including new and enlarging lesions of the cerebrum, optic nerves, optic chiasm, area postrema, brainstem, and cerebellum
• Contrast-enhancing T1-weighted lesions of the cerebrum, optic nerves, optic chiasm, area postrema, brainstem, and cerebellum; optional meningeal enhancement
• Global and regional brain volume loss, including basal ganglia, cerebellum, and upper cervical spinal cord
**MRI assessment of the spinal cord will involve:**
• New and persisting short tau inversion recovery or proton density hyperintense lesions and T1-weighted contrast enhancement (qualitative neuroradiological assessment)

MRI, magnetic resonance imaging.

### 2.9. Cognitive assessments

The Symbol Digit Modalities Test (SDMT) will be used to detect impairment of key neurocognitive functions that underlie many substitution tasks, including sustained attention, visual scanning, and recent memory.

### 2.10. Laboratory and biomarker assessments and mechanistic studies

Blood and CSF samples will be taken at the site during clinic visits and analyzed for hematology, serum chemistry (including liver enzymes, fibrinogen, and complement), AQP4-IgG, the PK of satralizumab, pharmacodynamic target engagement biomarkers (IL-6 and IL-6R), anti-satralizumab antibodies, and anti-RTX antibodies at baseline (Cohort 2 only).

Additional biomarker assessments may include: blood autoantibody titers (including AQP4-IgG); blood immunoglobulin levels (total Ig, IgG, IgM, and IgA isotype); blood and/or CSF soluble markers of CNS injury and glial biology (NfL, GFAP, sTREM2, S100B, Tau); blood immune/inflammatory markers (CXCL13, IL-17A); CSF immune profiling (deep molecular profiling using gene expression [scRNA sequencing], immune repertoire [B- and T-cell receptors] at single-cell level) and peripheral blood mononuclear cell (PBMC) immune profiling (deep molecular profiling [B- and T-cell subsets, monocyte and neutrophil subsets], gene expression [scRNA sequencing], immune repertoire [B- and T-cell receptors] at single-cell level).

### 2.11. PRO assessments

PRO instruments will be used to capture each participant's experience with satralizumab. The SymptoMScreen instrument will be used to assess symptom severity in 12 distinct neurological domains: mobility, dexterity, spasticity, body pain, sensation, bladder function, fatigue, vision, dizziness, cognition, depression, and anxiety (21). The Treatment Satisfaction Questionnaire For Medication (TSQM) II instrument, which has been validated in patients with chronic disease (22), will be used to evaluate patient satisfaction with satralizumab treatment. The impact of NMOSD on each patient's ability to work and perform regular activities will be assessed using the Work Productivity and Activity Impairment Questionnaire: General Health (WPAI: GH).

### 2.12. Safety assessments

Selected AEs for satralizumab include infections (including serious infections and opportunistic infections) and injection reactions (AEs that occur during or within 24 hours after study drug administration and are judged to be related to study drug injection).

AEs will be summarized by system organ class and preferred term based on Medical Dictionary for Regulatory Activities coding, and graded according to severity [from 1 (mild) to 5 (death)]. All deaths that occur during the protocol-specified AE reporting period, regardless of relationship to study drug, will be recorded.

### 2.13. Study endpoints

The efficacy endpoints that will be assessed in SAkuraBONSAI are shown in Table 3. Secondary (imaging) endpoints include MRI and OCT outcomes analyzed by cohort and visit. Exploratory endpoints will include SymptoMScreen, TSQM II, and WPAI: GH up to Week 96 (absolute scores and change from baseline, respectively). Adherence to satralizumab treatment will be checked via the structured telephone interview conducted by the site personnel on the following working day after every self-administration of satralizumab.

**Table 3:** Efficacy endpoints in SAkuraBONSAI.

**Primary efficacy endpoint:**
• Time to first relapse up to Week 96
**Other efficacy endpoints:**
• Proportion relapse-free after Week 96
• Annualized relapse rate after Week 96
• Severity of relapses
• Absolute EDSS score and change in EDSS from baseline up to Week 96
• Time to onset of cumulative disability progression sustained for ≥12 and ≥24 weeks
• Changes in visual acuity, and vision-related quality of life (NEI VFQ-25)
• Patient cognition (SDMT)

EDSS, Expanded Disability Status Scale; NEI VFQ-25, The National Eye Institute Visual Function Questionnaire-25; SDMT, Symbol Digit Modalities Test.

CSF and blood autoantibody titers, soluble biomarkers of immune activity and CNS injury, and cellular immune profiling measures, will be reviewed up to Week 96 (absolute values and change from baseline, respectively). PK analyses will calculate correlation coefficients of satralizumab concentrations in CSF and serum and various covariates of interest, such as biomarkers and selected efficacy and safety endpoints at different timepoints. Immunogenicity analyses will report the incidence of anti-satralizumab antibodies by visit and by cohort using descriptive statistics.

Safety variables to be assessed are AEs, AEs of special interest, serious AEs, injection-site reactions, participant withdrawals due to AEs, measurements of laboratory parameters, and vital signs (including body weight).

### 2.14. Statistical analyses

The rarity of NMOSD imposes severe restrictions on recruitment, so a sample size of 100 participants, including 60 participants in Cohort 1 and 40 in Cohort 2, was chosen based on feasibility. The analyses of this study are exploratory and will primarily make use of descriptive statistical methods. No formal confirmatory hypothesis test will be conducted, and no adjustment for multiple testing will be made. All endpoints will be assessed separately for each cohort.

The intent-to-treat (ITT) and safety populations will include all enrolled participants who received ≥1 dose of satralizumab. The per-protocol population will be used for supportive efficacy analyses and will include all ITT participants without major protocol deviations deemed to potentially affect the efficacy endpoints.

Time-to-first-event analyses will be performed using Kaplan–Meier estimates. Correlations between biological measures (CSF, serum, and/or plasma), imaging measures (MRI and OCT), and clinical measures (EDSS, relapse, cognition, visual acuity, and functioning) over different timepoints will be calculated using Spearman's correlation coefficient. For continuous endpoints, the mean, median, standard deviation, quartiles, minimum, maximum, and number of missing values will be presented by visit and cohort. For categorical variables, percentages and number of missing values will be presented by visit and cohort. OCT statistical analyses will follow the APOSTEL 2.0 recommendations (19). No formal interim analyses are planned.

Exploratory analyses of selected endpoints may be performed during the course of the study, for example, after all participants have completed the first 12 months of the treatment phase and the necessary data are available.

## 3. Discussion

SAkuraBONSAI, a Phase 4, prospective, open-label study in patients with AQP4-IgG+ NMOSD, will investigate the efficacy and safety of satralizumab in newly diagnosed, treatment-naïve patients, and in patients who have responded inadequately to RTX.

A clinical study in NMOSD offers an opportunity to further understand the underlying pathophysiology of the condition and potential mechanisms of action of treatments. SAkuraBONSAI will use MRI, OCT, and biomarker analyses to evaluate possible prognostic markers of relapse and predictive markers of treatment efficacy. The study will also explore in detail the mechanism of action of satralizumab and the immunological and neurological changes following treatment in NMOSD. PROs will be used throughout the study to improve our understanding of the impact of NMOSD on people's quality of life, work, and activities of daily living.

With diagnostic criteria established (4), there remains a need to identify markers of disease activity and predict periods of relapse in patients with NMOSD. Immunological and neurological biomarker data generated from concurrent blood and CSF samples from many participants will provide valuable insights into the inflammation and neurodegeneration mechanisms in the pathophysiology of NMOSD, and paraclinical markers such as MRI and OCT can provide useful information for disease monitoring (18). SAkuraBONSAI will take a combined approach to evaluating the mechanistic characteristics of patients with NMOSD during the early stages of their condition, using longitudinal clinical, neurological, immunological, and imaging biomarkers to efficiently monitor the evolution of patients' disease over time with satralizumab treatment. Around a quarter of newly diagnosed, treatment-naïve patients experience lasting disability after failing to respond to rescue therapy following their first attack (23). The findings of SAkuraBONSAI will advance our understanding of new-onset NMOSD and may yield information that improves the speed and accuracy of treatment decisions, ultimately minimizing disability following a first attack. However, relapse events are predicted to be rare in SAkuraBONSAI due to the efficacy of satralizumab in AQP4-IgG+ NMOSD (6, 7, 24). As such, the dataset produced by relapsing patients is expected to be limited, restricting the scope of analyses into potential predictors of attacks.

A unique feature of SAkuraBONSAI is that MRI and OCT will be assessed at regular intervals, independently of symptoms. Changes in MRI (T2w and CE-T1w lesions) and OCT (GCIPL thinning) have been reported in NMOSD subjects independent of clinical attacks (25, 26). Regular OCT and MRI imaging in a prospective cohort of AQP4-IgG+ NMOSD subjects with detailed clinical, laboratory, visual, and imaging assessments will allow us to investigate the underlying hypothesis that inter-attack changes in OCT and MRI imaging in NMOSD patients correlates with subclinical measures of inflammatory injury. Such information will improve our understanding of NMOSD pathology and treatment efficacy, and facilitate the development of therapeutic biomarkers. Imaging analyses may provide additional insight into whether geographic lesion localization is directly related to future risk of disability. There are recent reports of patients presenting with optic neuritis only having slower disability accrual than patients with transverse myelitis only, whereas area postrema lesions may be associated with higher risk of disability (27).

A future goal in the management of NMOSD is to be able to characterize a patient's prognosis in the early phases of disease and predict treatment response (18). Some studies show that NfL and GFAP are elevated in patients with NMOSD, particularly during periods of disease activity (15–17). An analysis using data from the Phase 2/3 N-MOmentum study found that 62 out of 215 patients with NMOSD (29%) had elevated GFAP concentrations at baseline, and that these individuals had a 3-fold increased risk of relapse vs. patients with normal GFAP levels (15). GFAP levels were found to be significantly elevated within 1 week of an attack (from 168 to 2,160 pg/ml) and correlated with attack severity (15). Results from N-MOmentum also showed that high levels of serum GFAP were associated with subclinical MRI activity in individuals who did not relapse (15); this relationship will be further evaluated prospectively in SAkuraBONSAI. The longitudinal assessment of NfL and GFAP levels may be valuable for monitoring treatment response in NMOSD. In a single-center study of patients with NMOSD in China, treatment with RTX or the IL-6R antagonist tocilizumab reduced plasma levels of NfL and GFAP compared with oral corticosteroids, with NfL levels decreasing to the levels of healthy controls (17). RTX treatment reduced NfL and GFAP plasma levels by 21 and 23%, respectively, and tocilizumab treatment reduced NfL and GFAP plasma levels by 35 and 36%, respectively (17).

In contrast to multiple sclerosis, the role of MRI in NMOSD disease management is not well established. A number of conventional MRI measures have been identified for their prognostic value, with atrophy associated with poor recovery, chronic pain, and lasting disability (28). Some individuals with AQP4-IgG+ NMOSD continue to relapse despite receiving maintenance treatment that is effective in other patients. Patients with NMOSD who have been treated with RTX represent a significant proportion of the overall patient population (11), with some experiencing an inadequate clinical response to treatment (12, 13). Identifying predictors of treatment response would enable physicians to prescribe the most suitable treatment in a more accurate and timely manner. SAkuraBONSAI will evaluate and compare the baseline characteristics of inadequate responders to RTX and its biosimilars against newly diagnosed patients to explore how they respond to satralizumab. During the study, participants will be tapered off corticosteroid therapy in the screening period to reduce any transient corticosteroid treatment effect on baseline assessments, while minimizing any treatment-free period. Results from the SAkuraStar study have shown that satralizumab monotherapy is effective at reducing the risk of relapse (7), so reserving corticosteroids for use as rescue therapy during the study is feasible.

Treatment response to acute rescue therapy is also important in minimizing persistent disability in NMOSD. A retrospective study of 185 patients in the Neuromyelitis Optica Study Group (NEMOS) registry found that, following first-line treatment, 64.5% of treated attacks showed partial response and 16.4% showed no response; predictors of poor treatment response included age, presence of myelitis, and complete remission from previous attack, with therapy escalation associated with improved outcomes (29). The exploratory assessments in SAkuraBONSAI may help to identify the risk factors of an attack being resistant to first-line rescue therapy, helping physicians to initiate a treatment escalation with minimal disability accrued.

Data generated during baseline assessments may provide clinically relevant insights into the underlying pathophysiology of NMOSD in newly diagnosed patients and among those who have not responded to RTX, offering an opportunity to rapidly disseminate information generated during the cross-sectional part of the study. Data for treatment-related outcomes will be generated longitudinally and will be disseminated once the study has been completed.

In conclusion, the SAkuraBONSAI study offers an opportunity to develop a greater understanding of the natural history of NMOSD, including the potential to identify imaging and molecular biomarkers that may inform the prognosis and management of patients with newly diagnosed NMOSD, while understanding how patients who do not respond to RTX therapy compare with a treatment-naïve population. By following changes in imaging, OCT, and biomarker parameters over time in patients treated with satralizumab, greater insight will be gained into the mechanism of action of satralizumab therapy, its efficacy and safety in newly diagnosed patients with NMOSD, and patients who have not responded to RTX therapy, as well as evaluating potential predictive/prognostic biomarkers.

## Data availability statement

The original contributions presented in the study are included in the article/supplementary material, further inquiries can be directed to the corresponding author.

## Ethics statement

The study is being performed in compliance with the International Conference on Harmonization, in accordance with Good Clinical Practice guidelines, US Food and Drug Administration regulations and in line with the principles of the Declaration of Helsinki and applicable local, regional, and national laws. All participants are required to provide written informed consent before any study-related procedures, including screening evaluations, are performed. Institutional review board approval will be obtained at each participating center before activating each study site. The study was prospectively registered on ClinicalTrials.gov prior to enrolling the first participant (ClinicalTrials.gov identifier: NCT05269667). Results of this study will be reported periodically, as data become available, at international congresses where NMOSD is of interest to the audience and published in international peer-reviewed journals.

## Author contributions

VGA, RB, TK, ANL, and SL-C contributed to the methodology, software, validation, formal analysis, and visualization of the work. All authors contributed to the conceptualization, investigation, resourcing for the study work, drafting, revising, critically reviewing the manuscript for important intellectual content, and approved the final version of this manuscript to be published and agree to be accountable for all aspects of the work in ensuring that questions related to the accuracy or integrity of any part of the work are appropriately investigated and resolved.
